# Reference Values for and Correlation Analysis of the Anogenital Distance of Chinese Han Full-Term Singleton Neonates

**DOI:** 10.3389/fped.2022.905421

**Published:** 2022-06-02

**Authors:** Wei Cao, Xiaowei Ding, Zhiya Dong, Haiting Tang

**Affiliations:** ^1^Department of Pediatrics, Ruijin Hospital, School of Medicine, Shanghai Jiao Tong University, Shanghai, China; ^2^Department of Obstetrics and Gynecology, Ruijin Hospital, School of Medicine, Shanghai Jiao Tong University, Shanghai, China

**Keywords:** anogenital distance, full-term neonates, neonates, singleton, reference value

## Abstract

**Background:**

Anogenital distance (AGD) is a biomarker used for the evaluation of fetal androgen action. The disruption of fetal androgen action can affect the development of the reproductive system and adversely affect future reproductive functions. However, AGD may differ by race. Currently, there is a lack of data regarding the evaluation of AGD in large Han Chinese samples.

**Objective:**

AGD for neonates in Shanghai, China, was measured, and relevant factors that influenced AGD were analyzed.

**Methods:**

The AGD of full-term singleton neonates was measured within 3 days of birth, and the results were grouped according to gestational age and body weight at birth. In addition, relevant factors that influenced AGD were investigated.

**Results:**

A total of 1,867 full-term singleton neonates were enrolled in this study. All the neonates were Han Chinese; among them, 986 were male, and 881 were female. Male AGD was 23.18 ± 3.17 mm, and female AGD was 11.65 ± 1.53 mm. Male AGD was 1.99 times longer than female AGD. With the increase in gestational age and body weight, AGD gradually increased. AGD was correlated with gestational age, body weight, and head circumference. The correlation between body weight at birth and AGD was highly significant.

**Conclusion:**

This study, for the first time, reported AGD measurement data for Chinese Han neonates. The results indicated that AGD was correlated with gestational age, body weight, and head circumference. The correlation between body weight at birth and AGD was highly significant.

## Introduction

Anogenital distance (AGD) is the distance between the anus and the genitalia. It was originally used in rodents to distinguish between sexes. It exhibits sexual dimorphism, with male AGD being longer than female AGD (1). AGD is determined within a window of androgen action. Sex differentiation begins during this window, developing toward male phenotypes under the action of androgen. Exposure to estrogens or anti-androgen substances within the window can lead to abnormal genital development, resulting in reproductive malformations such as short AGD, hypospadias, and cryptorchidism (2, 3). At birth, AGD can be used as a non-invasive indicator of the level of masculinization and for predicting abnormal reproductive system development (4). The clinical and epidemiological use of AGD is increasing; however, reference data for AGD are scarce. This study aimed to provide AGD reference data for Chinese Han newborns and investigate relevant factors that influence AGD.

## Patients and Methods

### Study Participants

Chinese Han singleton neonates with a gestational age ≥37 weeks born in Shanghai, China, between January 2019 and December 2021 were enrolled. The exclusion criteria were as follows: mothers or neonates who had an incomplete medical history; neonates with combined severe malformations such as chromosomal diseases, critical congenital heart disease, or diaphragmatic hernia; neonates who were directly transferred to the neonatal intensive care unit for treatment due to other reasons; and neonates who presented genital malformations, hypospadias, or cryptorchidism. This study was approved by the Ethics Committee of Ruijin Hospital, Shanghai JiaoTong University School of Medicine. The guardians of all neonates were fully informed and signed an informed consent form.

### Methods

#### Measurement Methods

AGD was measured when neonates were asleep or in a calm state in a quiet and bright examination room with the room temperature maintained at ~25°C. Neonates were placed in a supine position with bilateral knee flexion to fully flex and abduct the hip and fully expose the perineum. An assistant secured the neonates in the above body position, and a professionally trained pediatrician performed the measurements and recorded the data. To minimize differences caused by measurements being taken by multiple personnel, all neonates were measured by the same pediatrician. Neonatal mobility was minimized during each measurement, and the pediatrician minimized skin contact at the measurement site as much as possible. A Vernier caliper was held in the right hand and slightly tilted toward the newborn's head. The measurement accuracy of the Vernier caliper used in this study was 0.05 mm. All measurements were completed within 72 h of birth.

#### Description of the Measurements

**Figure 1:**
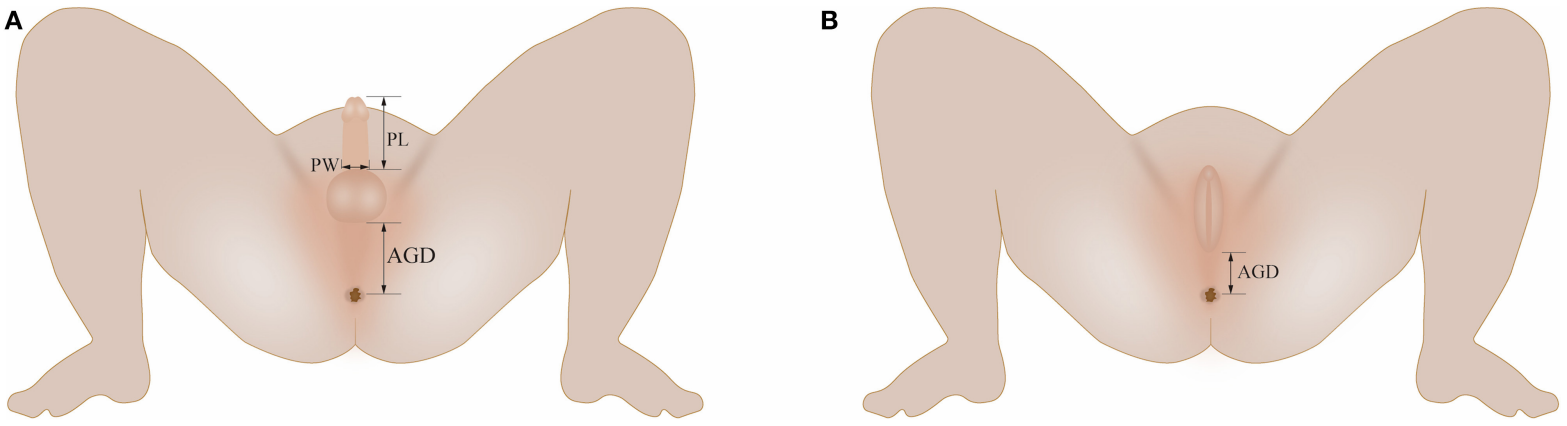
**(A)** Illustration of AGD, PL, and PW measurements in boys; **(B)** Illustration of AGD measurements in girls. AGD, anogenital distance; PL, penile length; PW, penile width.

The measurement method was performed in accordance with a previous literature report (5). Male AGD was the distance between the center of the anus and the base of the scrotum (Figure 1A). The base of scrotum was considered the first dermatoglyph of the scrotum at the junction of the scrotum skin and the perineal skin. If the scrotum needed to be lifted to find the junction of the perineum and the scrotum, excessive elongation of the perineum scrotum was avoided. Female AGD was the distance between the center of the anus and the posterior convergence of the labia (Figure 1B). Penile length (PL) was measured using a Vernier caliper from the superior margin of pubic symphysis to the end of the penis (Figure 1A). The penis was picked up and stretched slightly. The foreskin was slid down slightly to expose the urethral opening, and the distance between the base of the dorsal surface of the penis and the tip of the glans was measured. Penile width (PW) was measured using a Vernier caliper to determine the diameter of the penile base (Figure 1A).

### Data Collection

Maternal information included age, body weight, height, body mass index (BMI), gravidity, parity, smoking and drinking history, and education level. Neonatal data included gestational age, body weight, height, and head circumference. All data were acquired after the collection of a detailed medical history.

### Statistical Analysis

Statistical analysis was performed using SPSS 26.0 statistical software. Measurement data are expressed as $\bar{x}$ ±*s*. Comparison between two groups was performed using the *t*-test. A two-way analysis of variance was used to compare the AGD between different genders, different gestational ages, and different birth weights. To study the relationship between maternal and neonatal factors and AGD, Pearson correlation analysis and multiple linear regression analysis were performed using AGD; maternal age, body weight, height, and BMI; and neonatal gestational age, body weight at birth, length, and head circumference. Maternal gravidity, parity, and education level were regarded as ordered data and were analyzed using the Spearman correlation analysis. *P* < 0.05 indicated statistical significance.

## Results

### General Information

A total of 1,867 Chinese Han full-term singleton neonates (986 males and 881 females) were enrolled. The male AGD was 23.18 ± 3.17 mm, and the female AGD was 11.65 ± 1.53 mm; the male AGD was longer than the female AGD, and the difference was statistically significant (*t* = 98.211, *P* < 0.001). The male AGD was 1.99 times longer than the female AGD. AGD reference values for neonates with different gestational ages and for males and females are shown in Table 1. Two-way analysis of variance was performed on AGD between different genders and different gestational ages, and there was a statistically significant difference in AGD between different gestational ages (*F* = 17.409, *P* < 0.001). A separate effect analysis of AGD between different gestational ages was then performed. There were differences in AGD among male neonates of different gestational ages, and the difference was statistically significant, *F*_(4, 1, 857)_ = 21.098, *P* < 0.001, partial η^2^ = 0.043. And there were differences in AGD among female neonates of different gestational ages, but the difference was not statistically significant, *F*_(4, 1, 857)_=2.084, *P* = 0.080, partial η^2^ = 0.004. The AGD reference values for neonates with different body weights at birth and males and females are shown in Table 2. Two-way analysis of variance was performed on AGD between different genders and different body weights at birth, and there was a statistically significant difference in AGD between different body weights at birth (*F* = 111.963, *P* < 0.001). A separate effect analysis of AGD between different body weights at birth was then performed. The comparison of AGD between male and female neonates with different body weights at birth indicated differences, and the differences were statistically significant [*F*_(4, 1, 857)_ = 95.152, *P* < 0.001, partial η^2^ = 0.170 and *F*_(4, 1, 857)_ = 32.866, *P* < 0.001, partial η^2^ = 0.066, for male and female neonates, respectively]. The penis length of male neonates was 18.90 ± 2.96 mm, and the penis width was 9.84 ± 1.18 mm. And with the increase of gestational age and body weight, the penis length and penis width also tend to increase gradually (Tables 3, 4). We also provide a percentile table for reference (Table 5). Swan et al. (6) have proposed a new concept, anogenital index (AGI): the body weight standardized index of AGD [AGI =A GD/weight (mm/kg)], and found that using AGI as a parameter has a better correlation with age. Therefore, we also show this indicator in the results (Table 5). The male AGI was 6.99 ± 0.92 mm/kg, and the female AGI was 3.60 ± 0.40 mm/kg.

**Table 1:** AGD reference values for male and female neonates with different gestational ages (mm, $\bar{x}$ ±*s*).

**Gestational** **age (week)**	**Male**		**Female**		***F*** **-value**	***P*** **-value**
	**Number**	**AGD**	**Number**	**AGD**		
	**of people**		**of people**			
37~37^+6^	131	22.27 ± 2.64	95	11.32 ± 1.39	1089.685	<0.001
38~38^+6^	411	22.83 ± 3.10	371	11.48 ± 1.54	4100.641	<0.001
39~39^+6^	235	23.35 ± 3.06	218	11.79 ± 1.41	2463.839	<0.001
40~40^+6^	154	23.75 ± 3.34	143	11.91 ± 1.62	1697.393	<0.001
41~41^+6^	55	25.58 ± 3.42	54	12.20 ± 1.60	795.998	<0.001

**Table 2:** AGD reference values for male and female neonates with different body weights at birth (mm, $\bar{x}$ ±*s*).

**Body weight** **at birth (g)**	**Male**		**Female**		***F*** **-value**	***P*** **-value**
	**Number**	**AGD**	**Number**	**AGD**		
	**of people**		**of people**			
<2,500	13	19.32 ± 1.83	24	9.79 ± 1.35	151.816	<0.001
2,500~3,000	162	21.30 ± 2.41	185	10.49 ± 1.40	2002.332	<0.001
3,000~3500	481	22.84 ± 2.79	431	11.62 ± 1.15	5667.494	<0.001
3,500~4,000	275	24.33 ± 3.17	210	12.68 ± 1.29	3204.035	<0.001
≥4,000	55	26.83 ± 3.01	31	13.50 ± 1.47	698.477	<0.001

**Table 3:** PL and PW reference values for male neonates with different gestational ages (mm, $\bar{x}$ ±*s*).

**Gestational**	**PL**	**PW**
**age (week)**		
37~37^+6^	18.14 ± 2.88	9.47 ± 1.16
38~38^+6^	18.73 ± 2.82	9.77 ± 1.22
39~39^+6^	19.01 ± 2.91	9.86 ± 1.12
40~40^+6^	19.52 ± 3.04	10.08 ± 1.06
41~41^+6^	19.70 ± 3.60	10.44 ± 1.11

**Table 4:** PL and PW reference values for male neonates with different body weights at birth (mm, $\bar{x}$ ±*s*).

**Body weights**	**PL**	**PW**
**at birth (g)**		
<2,500	15.11 ± 2.33	8.03 ± 0.54
2,500~3,000	17.48 ± 2.95	9.10 ± 1.06
3,000~3,500	18.55 ± 2.67	9.74 ± 1.08
3,500~4,000	19.95 ± 2.79	10.31 ± 1.08
≥4,000	21.71 ± 2.32	10.87 ± 0.87

**Table 5:** Measurement parameter percentile table.

	**5th**	**10th**	**25th**	**50th**	**75th**	**90th**	**95th**
**Males**							
AGD (mm)	18.47	19.44	21.00	22.68	25.20	27.80	29.08
AGI (mm/kg)	5.59	5.83	6.30	6.93	7.62	8.22	8.57
PL (mm)	13.97	14.79	16.80	18.90	20.80	22.70	23.65
PW (mm)	7.87	8.29	8.99	9.90	10.65	11.42	11.80
**Females**							
AGD (mm)	9.40	9.85	10.55	11.65	12.60	13.45	14.10
AGI (mm)	3.04	3.15	3.38	3.59	3.80	4.00	4.19

### Univariate Analysis of AGD

The results of the correlation analysis of neonatal AGD and neonatal factors indicated that regardless of sex, neonatal AGD was positively correlated with neonatal gestational age, body weight at birth, body length, and head circumference (*P* < 0.001) (Table 6). The correlations between male neonatal PL and PW with AGD were positive and highly significant; the r values were 0.688 and 0.718, respectively, and the *P*-values were both <0.001. The results of the correlation analysis of AGD and maternal factors indicated that male AGD was correlated with maternal body height but was not correlated with maternal age, body weight, BMI, gravidity, parity, and education level. Female AGD was correlated with maternal body weight and BMI but was not correlated with maternal age, body height, gravidity, parity, and education level.

**Table 6:** Analysis of influencing factors associated with neonatal AGD.

**Factors**	**Male AGD**		**Female AGD**	
	***r*** **value**	***P*** **-value**	***r*** **-value**	***P*** **-value**
**Neonatal factors**				
Gestational age	0.226	<0.001	0.161	<0.001
Body weight at birth	0.319	<0.001	0.450	<0.001
Body length	0.460	<0.001	0.625	<0.001
Head circumference	0.270	<0.001	0.506	<0.001
**Maternal factors**				
Age	0.003	0.937	0.018	0.590
Body weight	0.069	0.031	0.183	<0.001
Height	0.119	<0.001	0.083	0.014
BMI	0.027	0.402	0.161	<0.001
Gravidity	0.016	0.608	0.076	0.025
Parity	0.050	0.116	0.027	0.422
Educational level	0.014	0.664	−0.027	0.418

### Multivariate Regression Analysis of AGD

Neonatal gestational age, weight at birth, body length at birth, and head circumference and maternal age, body weight, height, and BMI were used as independent variables, and AGD data were used as dependent variables for multiple linear regression analyses. The results of the analyses indicated that male and female neonatal gestational age, body weight at birth, and head circumference were correlated with neonatal AGD, of which the correlation between body weight at birth and AGD was highly significant (Table 7).

**Table 7:** Multiple regression analysis of AGD correlation in neonates.

**Factors**	**Male AGD**				**Female AGD**			
	**β value**	***95%CI***	**Standardized β value**	***P*** **-value**	**β value**	***95%CI***	**Standardized β value**	***P*** **-value**
Gestational age	0.249	0.052~0.446	0.079	0.013	−0.096	−0.184~-0.009	−0.063	0.032
Body weight at birth	0.004	0.004~0.005	0.538	<0.001	0.002	0.002~0.003	0.591	<0.001
Body length at birth	−0.226	−0.509~0.057	−0.067	0.118	−0.047	−0.169~0.075	−0.030	0.449
Head circumference	−0.264	−0.484~-0.044	−0.092	0.019	0.156	0.048~0.264	0.110	0.005
Maternal age	0.017	−0.026~0.060	0.023	0.434	−0.005	−0.024~0.013	−0.015	0.572
Maternal body weight	−0.062	−0.385~0.262	−0.187	0.708	−0.038	−0.173~0.096	−0.241	0.577
Maternal height	0.070	−0.159~0.299	0.113	0.548	0.023	−0.074~0.120	0.075	0.642
Maternal BMI	0.144	−0.715~1.003	0.151	0.742	0.104	−0.250~0.458	0.234	0.563

## Discussion

The development of the perineum and external genitalia is determined by dihydrotestosterone, resulting in a greater AGD in males than females (7). AGD is determined during the masculinization programming window (MPW) in the fetal period. Shorter AGD at birth indicates a reduction in intrauterine androgen exposure (8). Many studies have reported that AGD shortening in boys is associated with the development of hypospadias and cryptorchidism (4, 9, 10) and correlated with shorter PL, poorer semen quality, lower testosterone levels, and adult male infertility (4, 8, 11–13). As an indicator closely associated with testicular function and male reproductive capacity, AGD has been preliminarily applied in the clinical setting (14). Researchers in other countries use AGD as an indicator for the clinical evaluation of masculinization (15). Therefore, attention should be paid to the presence of other external genital abnormalities in male neonates with significantly shortened AGD and to the presence of abnormalities in testicular function and male reproductive ability in adulthood. Basic research has indicated that phthalates have estrogen mimetic or anti-androgen effects. During the MPW, intrauterine exposure to dibutyl phthalate or flutamide will damage the production or action of fetal androgen and decrease AGD, testicular weight, and PL. The sizes of all male reproductive organs are determined by androgen exposure in the MPW, whereas the growth of reproductive organs after birth is determined by androgen actions after birth (16). Therefore, for male neonates with a significantly shortened AGD at birth, attention should be paid to whether there is a history of exposure to endocrine disruptors during pregnancy. Such a medical history can affect future lifestyle. In particular, when a mother is ready to get pregnant again, she should avoid exposure to endocrine disruptors to reduce the possible impact on offspring.

In women, a longer AGD is thought to be a masculinization effect and is caused by the excessively high level of androgen or ectopic activation of androgen receptors. It has been shown that a longer AGD in women is associated with elevated testosterone levels (17, 18). Daughters of women with polycystic ovary syndrome have a longer AGD (18, 19), suggesting higher levels of fetal exposure to testosterone during pregnancy in women with polycystic ovary syndrome (18). Neonates with congenital adrenal hyperplasia have a longer AGD (20). In adult women, a longer AGD is associated with more ovary follicles, whereas a shorter AGD is associated with endometriosis (21, 22). Therefore, follow-up can be performed on neonates with a significantly elongated AGD to observe whether they have high androgen expression or other abnormal sexual development, so as to recognize diseases such as atypical congenital adrenal hyperplasia. These children should receive long-term follow-up to avoid high androgen-related diseases in adulthood.

Regardless of neonate sex, an abnormally shortened or elongated AGD can be suggestive of some diseases or of abnormal development and can be suggestive of endocrine disruptor exposure during pregnancy. AGD measurements are relatively simple and non-invasive. Post-natal AGD measurement shows that the presence of an abnormal AGD in neonates has great significance for the early detection of diseases associated with abnormal sexual development and for guiding mothers to avoid excessive exposure to endocrine disruptors. However, there are few studies on the normal range for AGD. Some studies in western countries have reported AGD measurement values (5, 23, 24). However, there are differences in the measurements between different ethnic groups, and there is still a lack of relevant data in East Asia. Therefore, our center performed a large-sample study to provide the normal AGD range for the Chinese Han population.

Through the measurement and analysis of the AGD of neonates within three d after birth, this study confirmed that AGD is different between males and females. Male AGD was 1.99 times longer than female AGD; male AGD was 23.18 ± 3.17 mm, and female AGD was 11.65 ± 1.53 mm. One Turkish study with full-term neonates indicated that female AGD was 10.3 ± 0.2 mm and male AGD was 23 ± 0.6 mm (23). One study with Mexican full-term neonates showed that female AGD was 11 ± 2 mm and male AGD was 21 ± 3 mm (5). The above results are similar to the findings from our study. However, the differences between some study results and our study results are slightly larger. One study in Great Britain with full-term neonates showed that the average female AGD was 9.1 ± 2.8 mm and the average male AGD was 19.8 ± 6.1 mm (24). One study of neonatal AGD in Ghana showed that male and female AGDs were 25.5 ± 5.1 mm and 13.6 ± 2.7 mm, respectively (25). One cross-sectional large-sample study in India showed that male and female AGDs in that population were 25.6 ± 3.1 mm and 15.4 ± 1.7 mm, respectively (26). Different studies have reported that the ratio between male neonatal AGD and female neonatal AGD ranges from 1.66 to 2.33 (5, 23–26). All study results indicate that male AGD is longer than female AGD. However, some results among different studies are different; these differences may be due to different measurement tools, measurement methods, and enrolled populations. Different ethnic groups have been enrolled in different studies. The studies by Romano-Riquer et al. (7) and Troisi et al. (27) showed that maternal hormone levels during pregnancy might be different in different ethnic groups and may influence fetal hormone levels, thus affecting AGD and causing differences among different ethnic groups.

This study also grouped the neonates based on different gestational ages and body weights at birth. The results indicated that with the increase in gestational age or body weight at birth, male and female AGDs both showed gradual increasing trends. There were statistically significant differences in AGD between male neonates at different gestational ages, as well as between male and female neonates at different birth weights, while there were differences in AGD between female neonates at different gestational ages, but the difference was not statistically significant, which may be due to the small number of samples in some groups. The total sample size in this study was large; therefore, the results could provide normal AGD reference values for East Asian neonates with different gestational ages and body weights at birth. Furthermore, the results of the correlation analysis of neonatal AGD and neonatal factors indicated that the AGD of all neonates was significantly positively correlated with neonatal gestational age, body weight at birth, body length, and head circumference. The results of the correlation analysis of AGD and maternal factors indicated that male AGD was positively correlated with maternal body height. Female AGD was positively correlated with maternal body weight and BMI; however, the correlation was weak. Therefore, multivariate regression analyses were performed. The results showed that gestational age, body weight at birth and head circumference of both male and female neonates were correlated with neonatal AGD, of which the correlation between body weight at birth and AGD was highly significant. These findings are similar to those results reported in previous studies (7, 23, 25, 26). Swan et al. (6) found that using AGI as a parameter had a better correlation with age. Therefore, after removing the effect of weight on AGD, perhaps AGI can also be used as one of the important parameters for children of different ages in long-term follow-up. Our study also found that PL and PW of male neonates also tended to increase with gestational age and body weight. In addition, the results from this study also showed that PL and PW were highly significantly correlated (positive) with AGD, findings that are similar to those reported by Thankamony et al. (24) and Alaee et al. (11). Therefore, it might be feasible to use AGD to evaluate the development of sexual characteristics of male children. The condition of gonadal development is generally regarded as an indicator of reproductive ability. Therefore, whether AGD can be used to evaluate male reproductive ability deserves further study and confirmation.

In summary, this was a large-sample study that included Chinese Han full-term singleton neonates; the aim was to obtain AGD reference values for different sexes, gestational ages, and body weights at birth. The results indicated that neonatal gestational age, body weight at birth, and head circumference were correlated with neonatal AGD, of which the correlation between body weight at birth and AGD was highly significant. This study had certain limitations. The study center was in Shanghai, China, and the living environments of mothers enrolled in this study were similar; therefore, the influence of environmental factors on mothers was not included. Furthermore, long-term follow-up was not performed on these neonates; therefore, longitudinal data were not obtained. AGD reference data of children of different ages should be obtained through prospective cohort studies in the future to better investigate the clinical significance of AGD.

## Data Availability Statement

The original contributions presented in the study are included in the article/supplementary material, further inquiries can be directed to the corresponding author/s.

## Ethics Statement

The studies involving human participants were reviewed and approved by the Ethics Committee of Ruijin Hospital, Shanghai JiaoTong University School of Medicine. Written informed consent to participate in this study was provided by the participants' legal guardian/next of kin.

## Author Contributions

WC, ZD, and HT contributed to conception and design of the study. WC drafted the manuscript and analyzed the data. XD and HT collected the patients information. ZD reviewed the manuscript and contributed to the literature overview. All authors contributed to manuscript revision, read, and approved the submitted version.

## Conflict of Interest

The authors declare that the research was conducted in the absence of any commercial or financial relationships that could be construed as a potential conflict of interest.

## Publisher's Note

All claims expressed in this article are solely those of the authors and do not necessarily represent those of their affiliated organizations, or those of the publisher, the editors and the reviewers. Any product that may be evaluated in this article, or claim that may be made by its manufacturer, is not guaranteed or endorsed by the publisher.
